# A comparative analysis of rod bipolar cell transcriptomes identifies novel genes implicated in night vision

**DOI:** 10.1038/s41598-018-23901-6

**Published:** 2018-04-03

**Authors:** Sasha M. Woods, Edward Mountjoy, Duncan Muir, Sarah E. Ross, Denize Atan

**Affiliations:** 1 Bristol Medical School, University of Bristol, Bristol, BS8 1TD UK; 2 MRC Integrative Epidemiology Unit, University of Bristol, Bristol, BS8 2BN UK; 3 Departments of Neurobiology and Anesthesiology and the Center for Pain Research, University of Pittsburgh, Pittsburgh, 15213-2536 USA

## Abstract

In the mammalian retina, rods and a specialised rod-driven signalling pathway mediate visual responses under scotopic (dim light) conditions. As rods primarily signal to rod bipolar cells (RBCs) under scoptic conditions, disorders that affect rod or RBC function are often associated with impaired night vision. To identify novel genes expressed by RBCs and, therefore, likely to be involved in night vision, we took advantage of the adult *Bhlhe23*^−/−^ mouse retina (that lacks RBCs) to derive the RBC transcriptome. We found that genes expressed by adult RBCs are mainly involved in synaptic structure and signalling, whereas genes that influence RBC development are also involved in the cell cycle and transcription/translation. By comparing our data with other published retinal and bipolar cell transcriptomes (where we identify RBCs by the presence of *Prkca* and/or *Pcp2* transcripts), we have derived a consensus for the adult RBC transcriptome. These findings ought to facilitate further research into physiological mechanisms underlying mammalian night vision as well as proposing candidate genes for patients with inherited causes of night blindness.

## Introduction

In the mammalian retina, rods and a specialised rod-driven signalling pathway mediate visual responses under scotopic (dim light) conditions. This pathway is exquisitely sensitive to light, since the rod phototransduction cascade is sensitive to single photons of light; and because 20–80 rods synapse with each rod bipolar cell (RBC) in the pathway, meaning that RBCs integrate rod signals over a wide receptive field^1^. Although there is only one type of rod and one type of RBC, rods are the most prevalent photoreceptors and RBCs are the predominant bipolar subtype in the mammalian retina^2^. As the retinal circuitry downstream from RBCs is also involved in photoptic (bright light) signalling, purely scotopic vision is mainly dependent on rod and RBC function. Therefore, disorders that affect rod or RBC function or survival are usually associated with impaired night vision.

Inherited causes of night blindness in humans are genetically and clinically diverse, and are usually diagnosed from recordings of retinal responses to light (electroretinograms; ERGs) under varying levels of illumination. The ERG a-wave is predominantly driven by rods (under scotopic conditions) and cones (under photopic conditions) and the b-wave by post-synaptic bipolar cells. While RBCs are functionally “ON” bipolar cells, since they depolarise in response to light, cone bipolar cells (CBCs) are either “ON” (depolarise to light) or “OFF” (hyperpolarise to light). Hence, the results of ERG recordings under varying levels of illumination can help to localise which cell type is at fault in the visual pathways.

Most inherited causes of night blindness are characterised by *progressive* photoreceptor degeneration that predominantly affects rods; also known as retinitis pigmentosa (RP). In the early stages of RP, ERG recordings show that scotopic a-wave responses are affected and b-waves may be smaller in amplitude due to the secondary effects of impaired photoreceptor function on downstream signalling. In contrast, congenital stationary night blindness (CSNB) refers to a group of largely *non-progressive* inherited retinal disorders causing impaired night vision (reviewed in Zeitz *et al*.^3^). As in RP, the rare Riggs-type of CSNB is associated with reduced scotopic a-wave amplitudes (sometimes with an additional reduction in b/a-wave ratio) because rod function is either primarily affected by the gene defect, or secondarily affected by RPE dysfunction. Indeed, certain mutations of genes expressed by rods or RPE cells, e.g. *GNAT1*, *PDE6B*, and *RHO*, cause CSNB, and other mutations cause RP^4–8^. In the Schubert-Bornschein type of CSNB (linked to genes that influence rod-RBC synaptic signalling), the a-wave is normal but b-wave amplitudes are reduced (known as an electronegative ERG). This type of CSNB is further subclassified into complete (cCSNB) and incomplete (icCSNB) forms: cCSNB is linked to genes expressed post-synaptically in rod and cone ON-bipolar dendrites (*NYX*, *TRPM1*, *GRM6*, *LRIT3*, *GPR179*) which affect ON-bipolar signalling, whereas icCSNB is linked to genes expressed pre-synaptically in photoreceptors (*CACNA1F*, *CABP4*, *CACNA2D4*) which affect ON- and OFF-bipolar signalling (hence, photopic responses are more severely affected in ERG recordings from patients with icCSNB vs cCSNB). Consequently, the results of ERG recordings help to make a clinical diagnosis in patients with night blindness and also indicate which cell types have been affected by the disorder and the likely candidate genes.

In many cases, candidate genes for inherited causes of night blindness were identified in animal models before they were linked to human disease. For example, knock-out mouse models of *GRM6* and *TRPM1* existed before mutations were identified in CSNB patients^9,10^. In ERG recordings of mice with *GRM6* and *TRPM1* gene defects, the scotopic and photopic a-waves are preserved while the b-waves are reduced, making them good candidates for cCSNB^9,10^. *GRM6* and *TRPM1* are not exclusively expressed by RBCs - they are expressed by all ON-bipolar subtypes – and retinal organisation and bipolar cell survival are unaffected by the gene mutations in these mouse models (reviewed in Zeitz *et al*.^3^). This contrasts with two other mouse models of night blindness, *Bhlhe23*^−/−^ mice^11^ and *Prdm8*^*eGFP*/*eGFP*^ mice^12^, which are functionally null mutations of the transcription factor genes *Bhlhe23* (also known as BHLHB4 or basic helix-loop-helix family member, b4) and *Prdm8* (PRDI-BF1 and RIZ homology domain containing 8) respectively. In the *Bhlhe23*^−/−^ mouse retina, RBCs are almost completely absent, resulting in a thinner inner nuclear layer (INL) of the retina. Scotopic b-wave amplitudes are significantly reduced in ERG recordings from *Bhlhe23*^−/−^ mice (and photopic b-waves are also slightly reduced) while rod and cone driven a-waves are preserved^11^. Similarly, RBCs are nearly absent from the adult retina of *Prdm8*^*eGFP*/*eGFP*^ mice, as well as cone type 2 OFF-bipolar cells, resulting in a thinner INL^12^. In this model, scotopic and photopic b-wave amplitudes are reduced in ERG recordings, while the a-waves are preserved^12^. As both the *Bhlhe23*^−/−^ and *Prdm8*^*eGFP*/*eGFP*^ models have non-progressive electronegative ERG phenotypes, they are similar to the phenotypes of patients with the Schubert-Bornschein type of CSNB^11,12^, but the expression pattern of *Bhlhe23* in RBCs^11^ and PRDM8 in RBCs and subsets of CBCs, amacrine cells and ganglion cells^12^ does not exactly match the predictions of the complete vs incomplete subclassification of Schubert-Bornschein CSNB. Mutations in *BHLHE23* and *PRDM8* have not been identified in patients with CSNB to date – possibly due to the expression of these genes outside the retina in the CNS^13,14^ or because they are transcription factor genes that regulate the development and survival of specific retinal bipolar subtypes, rather than their function. Nevertheless, the *Bhlhe23*^−/−^ and *Prdm8*^*eGFP*/*eGFP*^ mouse models show that genetically-determined cellular defects involving RBCs cause an ERG phenotype resembling CSNB^11,12^. It is, therefore, possible that mutations in other RBC genes that affect RBC function or survival will result in night blindness and that further subclassifications of this disorder might be needed in future to better reflect the different possible pathogenetic mechanisms that can lead to night blindness.

Recent studies have taken advantage of technological innovations in gene expression profiling to identify different retinal cell types by their RNA transcriptomes with the aim to produce a genetic profile for each retinal cell or subtype that distinguishes it from other cells. For example, one study labelled RBCs with a *Cabp5*-GFP construct (which labels RBCs and Types 3 and 5 CBCs) before dissecting labelled cells from the developing retina at postnatal day 8 (PN8) and profiling individual bipolar subtypes with gene expression microarrays^15^. In another similar study, transgenic reporter mouse lines were used to label different cell types in the adult retina; these cells were isolated from whole retinal samples using fluorescence-activated cell sorting (FACS), then profiled with gene expression microarrays^16^. More recently, a novel method for high-throughput single-cell RNA-sequencing, Drop-seq, was used to analyse the transcriptomes of 44,808 mouse retinal cells at PN14, and different retinal cell types (neurons, glia, vascular cells) were identified post-hoc by clustering gene expression profiles^17^. Later, the same group used their Drop-seq technique to profile retinal bipolar cells (and Müller glia) after FACS of *Vsx2*-GFP labelled cells from the retina of a *Vsx2*-GFP transgenic mouseline at PN17^18^. These studies all have different biases related to differences in their methods; for example, RNA-sequencing vs microarrays, the specificity of reporter vectors and mouselines for RBCs, the identification of RBCs either pre- or post-hoc, and differences in the timepoints chosen for the studies. Thus, whilst all these studies confirm that RBCs are the most common bipolar cells in the mouse retina and there is only one type of RBC, there is still no consensus about what constitutes the RBC transcriptome. Moreover, the likely function of these genes has not been investigated.

Our aim in this study was to take advantage of the specific cellular defect in RBCs described in the *Bhlhe23*^−/−^ mouse retina^11^ to derive the adult RBC transcriptome. As BHLHE23 is specifically expressed by RBCs during retinal development and BHLHE23 is a transcription factor that is required for RBC survival^11^, we were also interested to know which genes are regulated by BHLHE23 and, therefore, likely to be important to RBC development and survival. We found that genes expressed by adult RBCs are mainly involved in synaptic structure and signalling, whereas genes that influence RBC development are also involved in the cell cycle and transcription/translation. By comparing our data with other published transcriptomes of fully differentiated bipolar cells (where we identify RBCs by the presence of *Prkca* and/or *Pcp2* transcripts)^16–18^, we have derived a consensus for the RBC transcriptome, and have investigated the expression of some of these novel genes in the retina. Since scotopic vision is mainly dependent on rod and RBC function, and defects in rod or RBC function or survival result in night blindness in animal models, it is likely that some of the genes we have identified in the developing and adult *Bhlhe23*^−/−^ retina will be implicated in patients with electronegative scotopic ERGs from inherited causes of night blindness.

## Results

### The adult rod bipolar cell transcriptome

We identified genes that were likely to be important to RBC maintenance and function by comparing the transcriptomes of adult *Bhlhe23*^−/−^ mouse retinas with wild type (WT) retinas using Affymetrix gene expression microarrays. We identified 125 probes corresponding to 84 genes that were significantly down-regulated in adult *Bhlhe23*^−/−^ retina compared to WT (n = 4 matched pairs) with an adjusted p-value (p_adj_) <0.05 (Supplementary Table S1). As expected, *Bhlhe23* was one of the most significantly down-regulated genes in the *Bhlhe23*^−/−^ retina (down 2.79 fold, p_adj_ value = 7.50e^−6^, Supplementary Figure S1a) and the samples clearly segregated by condition (WT vs *Bhlhe23*^−/−^, Supplementary Figure S2a). Although there was a modest fold-change in *Bhlhe23* expression, the p_adj_ value for this difference in gene expression was very significant, reflecting the low baseline expression of *Bhlhe23*, since bipolar cells constitute only 8–10% of adult retinal cells and RBCs make up 38% of this fraction^19^. As p-values take the fold-change *between* conditions and the consistency of these differences *within* conditions into account, differentially expressed genes with significantly low p-values were given highest priority for further investigation (see below). Several other down-regulated genes in the *Bhlhe23*^−/−^ retina were established RBC markers, including *Prkca* (down 2.34 fold, p_adj_ value = 1.44e^−5^), *Car8 (*down 7.05 fold, p_adj_ value = 2.21e^−8^), and *Pcp2* (down 4.40 fold, p_adj_ value = 2.21e-8). Other genes, expressed by ON-bipolar cells but not specific to RBCs, were also significantly down-regulated; for example, *Trpm1*, Solute carrier family 5 member 8 (*Slc5a8*), Calcium channel, voltage-dependent, alpha 2/delta subunit 3 (*Cacna2d3*), and A Disintegrin-like And Metallopeptidase (reprolysin type) with ThromboSpondin type 1 motif, 5 (aggrecanase-2) (*Adamts5*)^20^. Hence, we were confident that our methods had identified both known and novel genes that were expressed by adult RBCs.

### The adult rod bipolar cell transcriptome regulates synaptic structure and signalling

**Figure 1:**
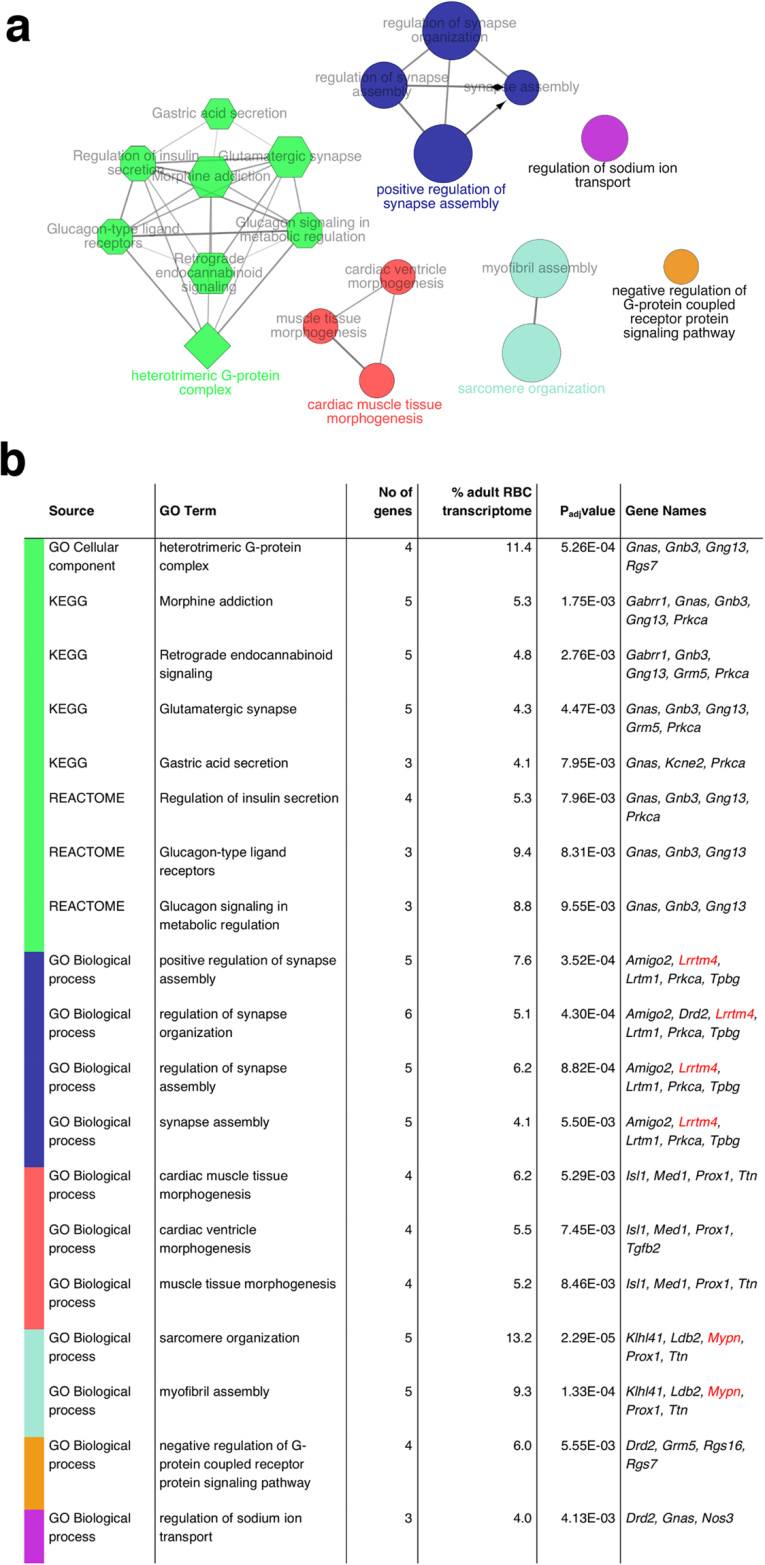
The adult rod bipolar cell transcriptome regulates synaptic structure and signalling. Gene ontologies and biological pathways involved in cell signalling and synaptic assembly are functionally enriched in the adult RBC transcriptome with p_adj_ ≤ 0.25. (**a**) Figure showing enriched gene ontologies cluster into 6 groups that share >50% of genes. Node size represents the statistical significance of enrichment and node shape corresponds to ontology source: circle = Gene ontology (GO) BiologicalProcess; diamond = GO CellularComponent; hexagon = Kyoto Encylopedia of Genes and Genomes (KEGG) pathway; octagon = REACTOME. (**b**) Table showing enriched gene ontologies and biological pathways in the adult RBC transcriptome, the corresponding gene names and the percentage (%) of the total number of genes in the adult RBC transcriptome associated with the Gene Ontology (GO) term. Colours correspond to functional groups in a. Genes selected for further investigation by immunolabelling/RT-PCR are in red.

We next asked what the adult RBC transcriptome could tell us about the genes that are responsible for normal RBC maintenance and function. To increase our power to detect enriched ontologies and pathways in these functional enrichment analyses, we accepted a false discovery rate adjusted p-value (p_adj_) of 0.25. Enrichment tests are still valid if the gene list contains false positives and because the inclusion of more (random) false positive terms would attenuate the enrichment test statistics in an unbiased way (bias towards the null). We thereby identified twelve gene ontology terms and seven biological pathways among the 131 most down-regulated genes (Supplementary Table S1). These genes clustered into six groups (Fig. 1a).

The largest functional cluster (8 genes) comprised of genes responsible for components of heterotrimeric G-protein complexes: Guanine nucleotide binding protein, alpha stimulating (*Gnas*), Guanine nucleotide binding protein, beta polypeptide 3 (*Gnb3*), Guanine nucleotide binding protein, gamma 13 (*Gng13*), Regulator of G-protein signalling 7 (*Rgs7*), Gamma-aminobutyric acid type A receptor Rho1 subunit (*Gabrr1*), Glutamate metabotropic receptor 5 (*Grm5*), Potassium Voltage-Gated Channel Subfamily E Regulatory Subunit 2 (*Kcne2*) and *Prkca* (Fig. 1b). These complexes are known to be present in glutamatergic synapses and retrograde endocannabinoid signalling pathways - including the glutamatergic rod-RBC synapse in the retina – where they act like a molecular switch to trigger intracellular signalling cascades in response to the activation of G-protein-coupled receptors.

A number of these genes (*Rgs7*^21^, *Grm5*^22^ and *Prkca*^23^) are also associated with insulin secretion and glucagon signalling in the pancreas, where BHLHE23 is also expressed^14^. Here, it is likely that BHLHE23 regulates the expression of some of the same genes in the pancreas and retina because of their common role in cell signalling.

The second largest gene cluster (6 genes) contained genes involved in the regulation of synapse assembly and organisation: Adhesion molecule with Ig like domain 2 (*Amigo2*), Dopamine Receptor D2 *(Drd2)*, Leucine Rich Repeat Transmembrane neuronal 4 *(Lrrtm4)*, Leucine-rich repeats and transmembrane domains 1 (*Lrtm1*), *Prkca* and Trophoblast glycoprotein *(Tpbg*)(Fig. 1b). Except for *Prkca* (see above) and *Drd2* (which is expressed in dopaminergic ACs), the cell-specific expression pattern of these genes in mammalian retina was not known.

### Genetic regulators of rod bipolar cell development

Our next aim was to determine which genes were differentially expressed in the developing *Bhlhe23*^−/−^ retina at PN7, just prior to the onset of RBC death^11^, so that we might identify important regulators of RBC development and survival.

**Figure 2:**
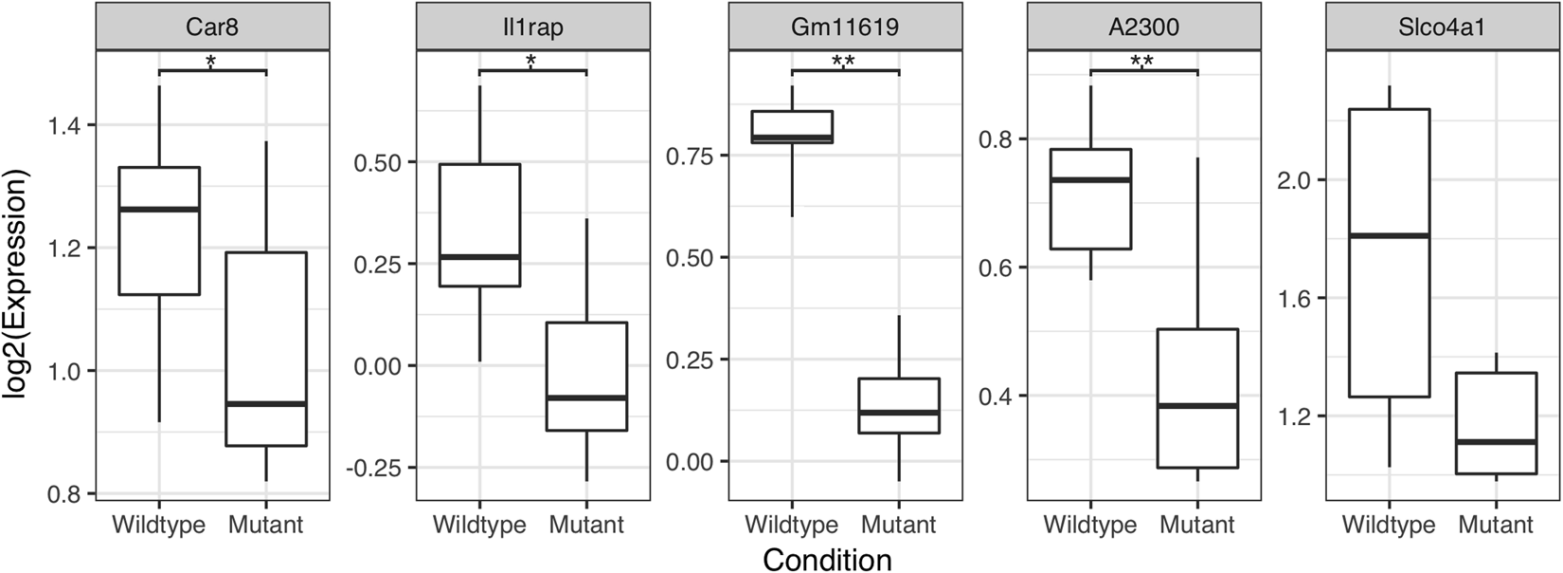
Several novel genes are differentially expressed in the *Bhlhe23*^−/−^ retina at postnatal day 7. Representative box plots showing the results of RT-PCR experiments to confirm the differential expression of novel genes (identified by RNAseq) in the *Bhlhe23*^−/−^ retina vs wildtype (WT) at PN7 (n = 6 litter-matched pairs). Four genes that were significantly down-regulated were *Car8 (p-value *=* 0*.*018)*, *Il1rap (p-value *=* 0*.*024)*, *Gm11619 (p-value *=* 8*.*6e*^−*4*^*) and A230077H06Rik* (p-value = 8.2e^−3^). The mean expression of *Slco4a1* was lower in the *Bhlhe23*^−/−^ retina vs WT but did not reach statistical significance (p-value = 0.069).

We found twenty-eight genes were mis-expressed in the *Bhlhe23*^−/−^ retina compared to WT at PN7 with a p_adj_ value < 0.05 using sex as a co-variate (Supplementary Table S1), of which 16 genes were down-regulated and 12 genes were up-regulated. As expected, *Bhlhe23* was the most significantly down-regulated gene in the *Bhlhe23*^−/−^ retina (down 119 fold, p_adj_ value = 5.7e^−105^, Supplementary Figure S1b), although the samples did not segregate as distinctly by condition (WT vs *Bhlhe23*^−/−^) and by sex as for the adult microarray experiment (Supplementary Figure S2b); most likely because only small differences in gene expression existed between conditions before the onset of RBC death. To confirm the differential expression of a subset of these genes in the PN7 *Bhlhe23*^−/−^ retina that we knew to be novel (compared with published literature), we selected those with the lowest p_adj_ values and highest fold changes (Supplementary Table S1) for validation by RT-PCR. We found that *Car8 (p-value* = *0*.*018)*, *Il1rap* (p-value = 0.024), Predicted gene 16119 (*Gm11619*) (p-value = 8.6e^−4^), and RIKEN cDNA A230077H06 gene (*A230077H06Rik*) (p-value = 8.2e^−3^) were significantly down-regulated in *Bhlhe23*^−/−^ retina compared to WT at PN7 by RT-PCR and weak evidence for the down-regulation of Solute carrier organic anion transporter family, member 4a1 (*Slco4a1*) (p-value = 0.069) (Fig. 2). Furthermore, the direction of our RT-PCR results was the same as our RNA-seq results, which again validated our methods.

### Regulators of RBC development are involved in synaptic signalling as well as transcriptional/translational activity and the cell cycle

**Figure 3:**
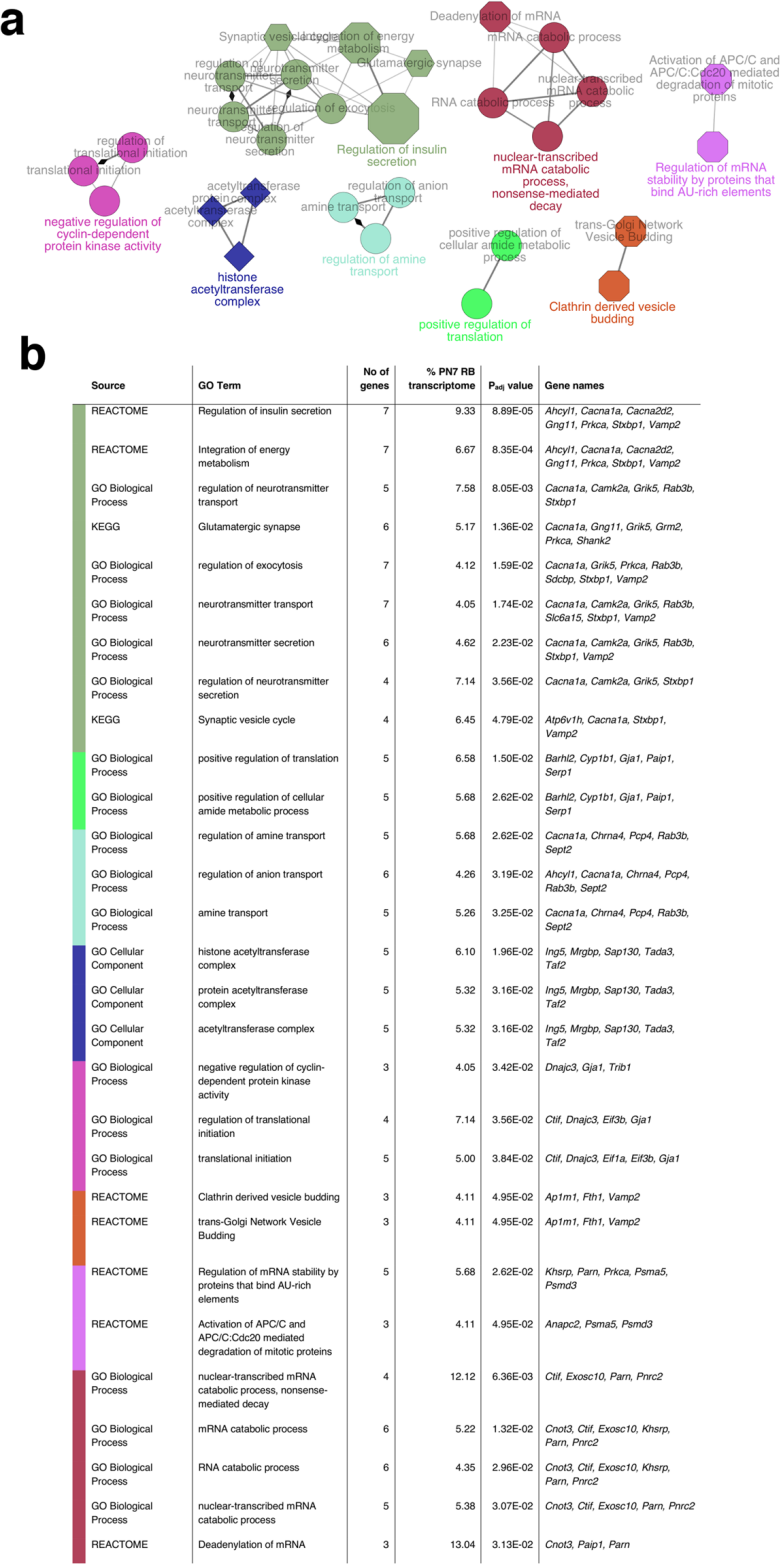
Regulators of rod bipolar cell development are involved in the cell cycle, synaptic signalling and transcriptional/translational activity. Gene ontologies and biological pathways involved in synaptic signalling, regulation of the cell cycle and transcriptional/translational activity are enriched in the *Bhlhe23*^−/−^ retina at PN7 with p_adj_ ≤ 0.25. (**a**) Figure showing enriched gene ontologies clustered into 8 groups that share >50% of genes. Node size represents the statistical significance of enrichment. Node shape corresponds to ontology source: circle = Gene ontology (GO) BiologicalProcess; diamond = GO CellularComponent; hexagon = Kyoto Encylopedia of Genes and Genomes (KEGG) pathway; octagon = REACTOME. (**b**) Table showing enriched gene ontologies and biological pathways in the *Bhlhe23*^−/−^ retina at PN7, the corresponding gene names and the percentage (%) of the total number of genes in the PN7 transcriptome associated with the Gene Ontology (GO) term. Colours correspond to functional groups in (**a**).

We next asked which gene ontologies and biological pathways were functionally enriched within the set of differentially expressed genes of the PN7 *Bhlhe23*^−/−^ retina. To increase the power of these analyses, we relaxed our FDR-adjusted p value to 0.25 as before, and found that 40 gene ontologies and biological pathways were enriched. Importantly, relaxing our FDR to this level meant that we included some key RBC genes, including *Prkca* and *Trpm1*. These genes clustered into eight main functional groups (Fig. 3a).

Like the adult RBC transcriptome, the largest gene cluster contained a total of 15 genes that were known to be involved in synaptic signalling and secretion - including insulin secretion - such as *Adenosylhomocysteinase Like 1* (*Ahcyl1*) and *G Protein Subunit Gamma 11* (*Gng11*) (Fig. 3a); however, there was little overlap in the identity of genes in these clusters between the developing and adult *Bhlhe23*^−/−^ retinal transcriptomes, since only *Prkca* was common to both (Figs 1b and 3b).

Predictably, there were more enriched genes involved in regulating gene transcription/translation and the cell cycle in the developing *Bhlhe23*^−/−^ retina compared with the adult RBC transcriptome. For example, *DnaJ heat shock protein family (Hsp40) member C3* (*Dnajc3*) is involved in the cell cycle; *Inhibitor of growth family*, *member 5* (*Ing5*), *MRG/MORF4L binding protein* (*Mrgbp*), *Sin3A associated protein* (*Sap130*), *Transcriptional adaptor 3* (*Tada3*) and *TATA box binding protein (TBP)-associated factor* (*Taf2*) regulate gene transcription; and *CBP80/20-dependent translation initiation factor* (*Ctif*), *Eukaryotic translation initiation factor 1* *A* (*Eif1a*) and *Eukaryotic translation initiation factor 3*, *subunit B* (*Eif3b*) regulate translation. We hypothesize that these genes are required for the proper differentiation and survival of RBCs during development, but are no longer needed for the normal maintenance and function of adult RBCs.

### Comparative analyses with other publically available datasets to derive a consensus for the adult RBC transcriptome

**Figure 4:**
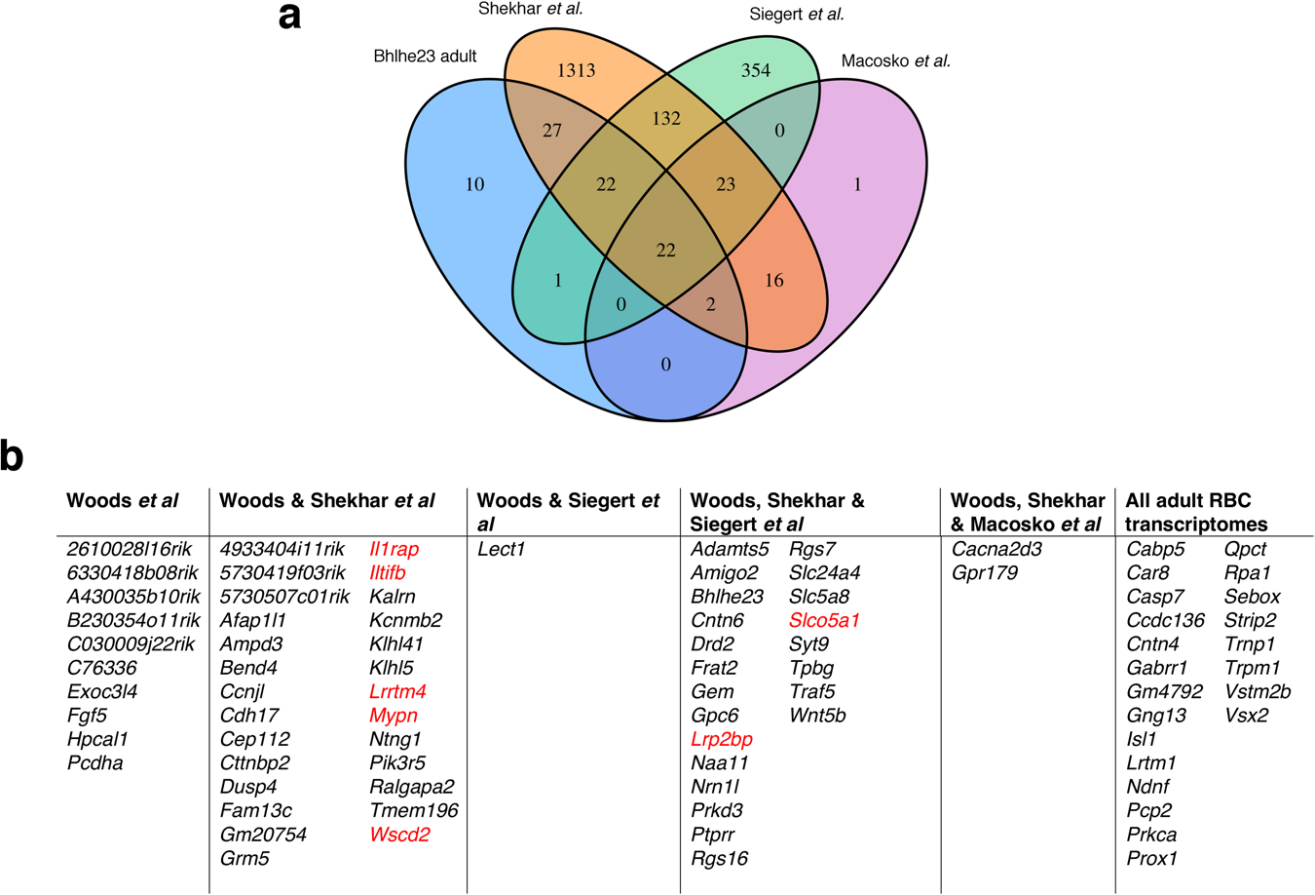
Comparative analyses of the adult RBC transcriptome with published datasets. (**a**) Venn diagram showing the degree of overlap between the datasets derived from the adult *Bhlhe23*^−/−^ retina (cited herein as Woods *et al*.) and other published transcriptomes^16–18^. The gene lists used to produce this plot and the degree in overlap are shown in Table S1. (**b**) Figure depicting the names of genes that overlapped between the datasets derived from the adult *Bhlhe23*^−/−^ retina (Woods *et al*.) and other published transcriptomes^16–18^. Genes are listed in alphabetical order and not in order of fold change or p_adj_ values. Genes selected for further investigation by immunolabelling are in red.

Of the 84 differentially expressed genes we identified as the adult RBC transcriptome (p_adj_ value < 0.05), 74 (88%) overlapped with other publically available adult RBC transcriptomes which we extracted from published datasets by identifying gene clusters in which *Prkca* and/or *Pcp2* were the most highly expressed^16–18^ (Fig. 4a and Supplementary Table S1).

Of the genes which overlapped between our own data (referred to as Woods *et al*.) and those published by others^16–18^, the majority were known to be RBC markers (*Cabp5*, *Car8*, *Casp7*, *Ccdc136*, *Cntn4*, *Isl1*, *Pcp2*, *Prkca*, *Prox1*, *Rpa1*, *Sebox*, *Trpm1*, *Vstm2b*, *Vsx2)* though few were specific to RBCs^15–18^ (Fig. 4b and Supplementary Table S1). Interestingly, there were a few examples of well-established RBC markers that were absent from some datasets, notably *Bhlhbe23* (from the Drop-seq data of different retinal cell types^17^) and *Prdm8* (from the microarray data of FACS-sorted cells from the *Pcp2*^+^ reporter mouseline^16^ and our own dataset) suggesting that all of these datasets need to be interpreted with caution. Our dataset shared the greatest overlap with the Drop-seq profiling of *Vsx2-GFP*^+^ retinal bipolar and Müller glial cells reported by Shekhar *et al*.^18^ where we identified RBCs by the *Prkca* transcript (73/84 genes overlapped); followed by the microarray data of FACS-sorted retinal cells from a variety of reporter mouselines published by Siegert *et al*.^16^ where we identified RBCs from the *Pcp2*^+^ reporter mouse line (45/84 genes overlapped). There were 10 genes we identified that were not found in any other available published dataset (Fig. 4b and Supplementary Table S1).

### Characterising the expression of novel RBC genes/proteins supports their likely roles in the synaptic signalling of adult RBCs

We next sought to confirm whether the novel genes and protein products we had identified in our adult RBC transcriptome were, indeed, expressed by RBCs and their localisation in RBCs using immunofluorescence microscopy. We again selected novel genes/proteins with the lowest p_adj_ values, greatest fold changes, and/or with multiple differentially expressed probe sets (indicated in red in Supplementary Table S1) that we had identified in our dataset and that overlapped with the two closest published RBC transcriptomes (Fig. 4b)^16,18^ and for which antibodies were available. We, therefore, selected the following genes for further investigation: *LRP2 binding protein* (*Lrp2bp*) (down 3.99fold, p_adj_ value = 3.90 × 10^−8^), Solute Carrier Organic Anion Transporter family, member 5A1 (*Slco5a1*)(down 2.88fold, p_adj_ value = 1.11 × 10^−6^), *Lrrtm4* (down 2.73fold, p_adj_ value = 7.81 × 10^−5^), *Il1rap* (down 4.72fold, p_adj_ value = 3.9 × 10^−8^), *Interleukin 10-related T cell-derived inducible factor beta* (*Iltifb*) (down 2.35fold, p_adj_ value = 2.27 × 10^−5^), *Myopalladin* (*Mypn*) (down 2.31fold, p_adj_ value = 1.53 × 10^−4^) and *WSC domain containing 2* (*Wscd2*) (down 4.04fold, p_adj_ value = 1.41 × 10^−7^)(Fig. 4b, Supplementary Table S1). We identified RBCs using antibodies directed toward the main RBC marker, PKCα (encoded by the gene, *Prkca*).

**Figure 5:**
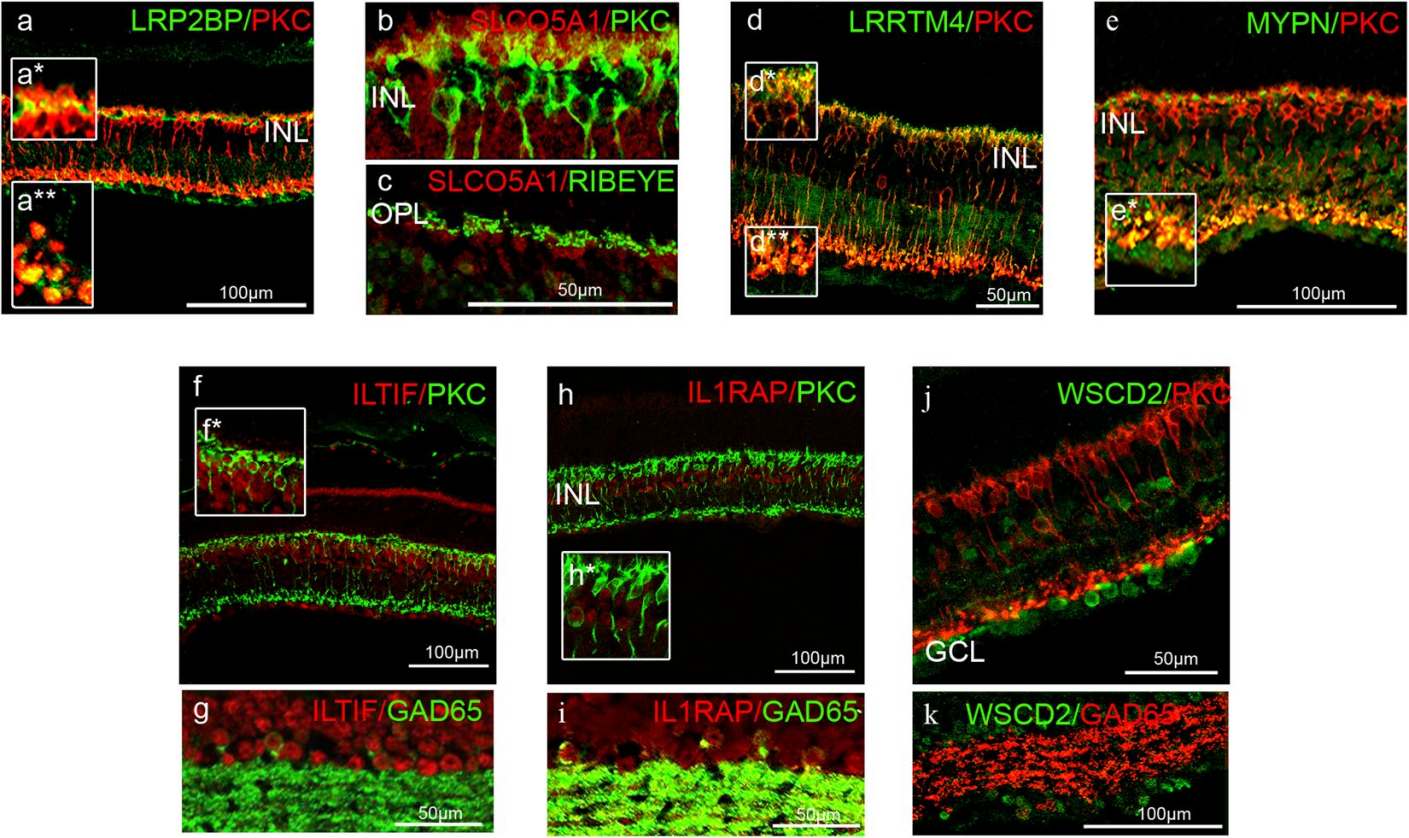
Characterisation of the expression of novel genes/proteins identified in the adult rod bipolar cell transcriptome. Representative confocal micrographs of adult wildtype (WT) retinal sections. LRP2BP **(a**,a*) SLCO5A1 **(b**–**c)** and LRRTM4 **(d**,d*) were expressed in PKC^+^ RBC dendrites. LRP2BP **(a**,a**) LRRTM4 **(d**,d**) and MYPN **(e**,e*) were also expressed in RBC axon terminals. **(f)** ILTIF was expressed in the nuclei of PKC^+^ RBCs, as well as **(g)** GAD65^+^ ACs. **(h**,**i)** IL1RAP and **(j**,**k)** WSCD2 were not expressed in PKC^+^ RBCs. (h*) IL1RAP was expressed in PKC^+^ ACs as well as **(i)** GAD65^+^ ACs. **(j)** WSCD2 was expressed in presumed ACs in the innermost INL as well as in cells of the GCL, but they were not GAD65^+^ **(k)**.

The genes *Lrp2bp* and *Slco5a1* were identified in the RBC transcriptome by our group and two others^16,18^ (Fig. 4b) and so they had a higher *a priori* probability of expression in RBCs. Both LRP2BP (Fig. 5a,a*) and SLCO5A1 (Fig. 5b,c) appeared to be localised to the dendrites of RBCs suggesting a role in synaptic signalling. In addition, LRP2BP appeared to be present in the large bulbous axon terminals of RBCs (inset, Fig. 5a**).

The genes *Lrrtm4*, *Il1rap*, *Iltifb*, *Mypn*, and *Wscd2* were identified in the RBC transcriptome by our group and one other^18^ (Fig. 4b, Supplementary Table S1). There was no previous information about the localisation of ILTIFB, MYPN, and WSCD2 expression in RBCs. Using an antibody that recognises both ILTIFA and ILTIFB isoforms, we found that ILTIF was expressed in the nuclei of PKC^+^ RBCs as well as presumed PKC^−^ CBCs and GAD65^+^ ACs in adult WT retina (Fig. 5f,f*,g); while MYPN, like LRP2BP, appeared to localise to the axon terminals of RBCs (Fig. 5e,e*). In contrast, we did not find that WSCD2 co-localised with PKC^+^ RBCs in WT adult retina (Fig. 5j), but appeared to be expressed in ACs in the INL and displaced ACs or ganglion cells in the ganglion cell layer (GCL); these ACs were GAD65^−^ (Fig. 5k).

Shekhar *et al*.^18^ reported that *Lrrtm4* and *Il1rap* transcripts were predominantly expressed in RBCs, and to a lesser extent in mixed populations of ACs, using fluorescent *in situ* hybridisation (FISH). We, too, found that LRRTM4 was expressed by PKC^+^ RBCs in the outer plexiform layer (OPL) (Fig. 5d), but in contrast to the nuclear localisation of *Lrrtm4* transcript, we found the protein to localise to the dendrites and axon terminals of RBCs (Fig. 5d*,d**). Additionally, we found that IL1RAP expression did not co-localise with PKC^+^ RBCs in adult WT retina (Fig. 5h). Instead, IL1RAP expression was clearly evident in the innermost INL of WT retina, where ACs cells are found. Some of these cells were GAD65^+^ ACs (Fig. 5i).

In summary, we found that LRP2BP, SLCO5A1, LRRTM4 and MYPN were expressed in the plexiform layers of the retina, supporting a role for them in synaptic signalling, and that ILTIF was expressed in the nuclei of RBCs, as well as CBCs and ACs. The expression of all five of these markers was down-regulated in *Bhlhe23*^−/−^ retina, consistent with their expression in RBCs (Supplementary Figure S3). Only SLCO5A1 and LRRTM4 appeared to be specific to RBCs while the expression of LRP2BP, MYPN and ILTIF appeared to persist in presumed CBCs (and ILTIF in ACs) (Supplemental Figure S3). In contrast to previous studies^18^, we found that IL1RAP and WSCD2 were expressed in ACs rather than RBCs in WT retina.

### Relatively few genes are exclusively expressed by rod bipolar cells

**Figure 6:**
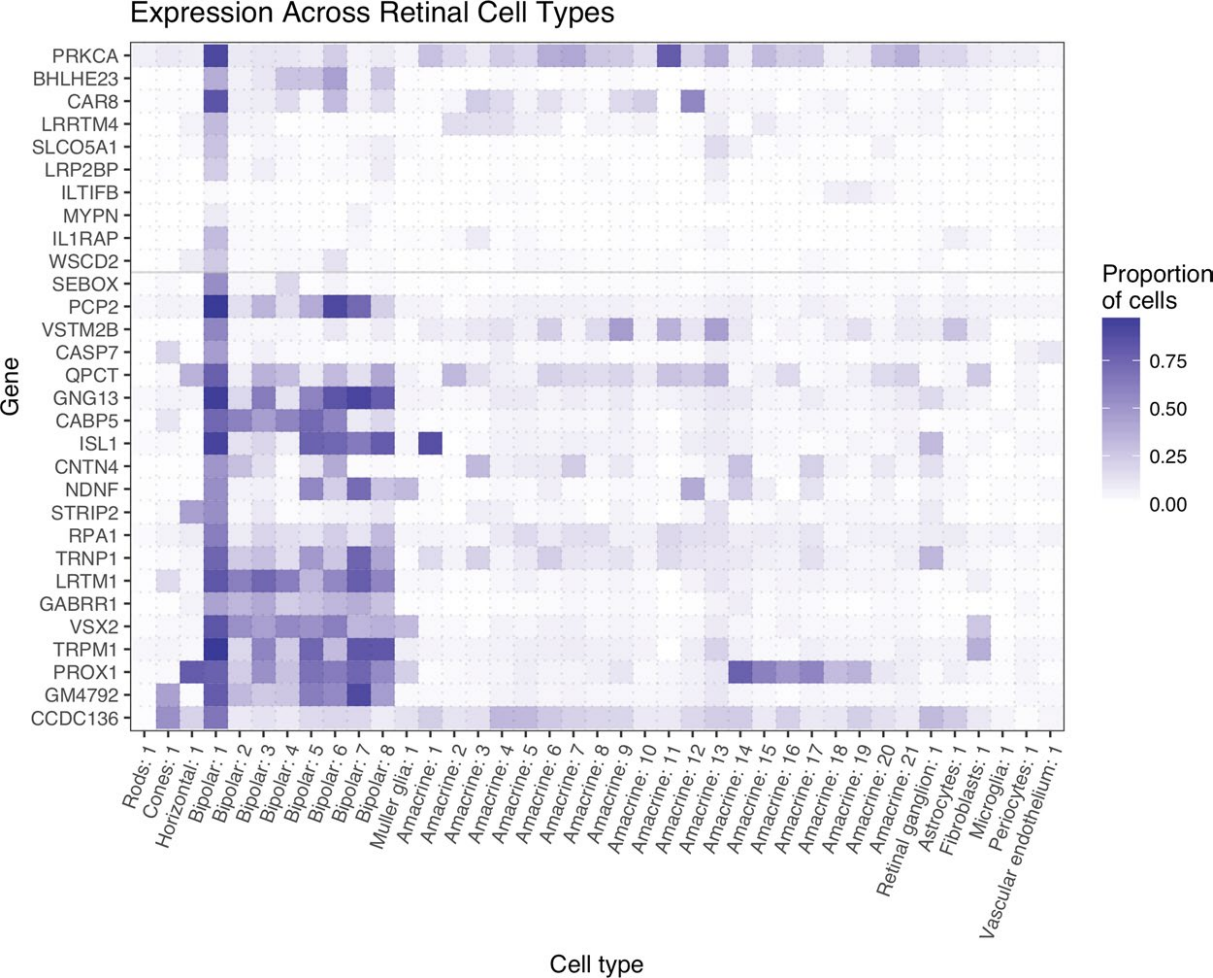
Relative expression of genes identified in the adult rod bipolar cell transcriptome in other retinal cell types. Heat map showing relative expression value (normalized by row) for genes, identified in the adult RB transcriptome, in other retinal cell types using published Drop-seq data for the whole retina^17^. The upper section shows RB genes identified in the *Bhlhe23*^−/−^ mouse model; the lower section includes the 22 genes found in all 4 adult RBC transcriptomes (Fig. 4). Intensity of colour shows the total proportion of cells, for that cell type, which express the gene of interest. ‘Bipolar 1′ was inferred to represent RBCs due to the high expression of *Prkca*. The results show that *Car8*, *Lrp2bp*, *Slco5a1*, *Lrrtm4*, *and Mypn* were all strongly expressed in the cluster identified as RBCs, although none were exclusive to RBCs. *Bhlhe23* is an established marker of RBCs but was not specifically expressed by RBCs in the Drop-seq profiling data of different retinal cell types^17^. In addition, *Iltifb* was expressed only at low levels in RBCs identified in the Drop-seq profiling data of different retinal cell types^17^, but occurred in the rod bipolar transcriptome we identified in our study and the Drop-seq profiling data of different bipolar subtypes^18^ (see Fig. 4b). Our results for IL1RAP and WSCD2 immunolabelling (Fig. 5i–k) suggest that these transcripts are either expressed at a low level in RBCs, not transcribed and/or the RBC data from Drop-seq profiling of different retinal cell types published by Macosko *et al*.^17^ was contaminated by a small number of ACs.

The discrepancy between the expression of *Il1rap* and *Wscd2* transcripts in RBCs and their protein products in ACs, suggested that some transcripts identified in the adult rod bipolar transcriptome were either not transcribed or their protein products were expressed in RBCs at relatively low levels compared with other retinal cell types. It also raised the possibility that some of the gene clusters derived from Drop-seq profiling of different retinal cell types^17^ were contaminated by >1 retinal cell type. Hence, we asked whether we could find evidence of the expression of genes identified in our RBC transcriptome in other retinal cell types, e.g. rods, cones, horizontal, ACs and ganglion cells, from the Drop-seq profiling data published by Macosko *et al*.^17^. We also looked for the gene transcripts of well-established markers of RBCs, such as *Prkca*, *Bhlhe23* and *Car8*, in other retinal cell types, as well as genes identified in our consensus RBC transcriptome (Fig. 6).

We found that even the transcripts of relatively well-established RBC-specific markers like *Prkca*, *Bhlhe23* and *Car8*, were expressed at low levels in clusters corresponding to other bipolar subtypes (CBCs) and ACs (Fig. 6). This finding suggested that that either these transcripts were not exclusive to RBCs and were expressed in low levels in other cell types, or that some of the gene clusters were contaminated by >1 retinal cell type. With regard to the novel rod bipolar genes we had investigated using immunolabelling, most appeared to be relatively unique to RBCs. *Iltifb* was expressed at very low levels in bipolar cluster 1 (corresponding to RBCs) as well as some amacrine clusters, suggesting that the depth of sequencing of the Drop-seq data was low. The presence of *Il1rap* and *Wscd2* transcripts in bipolar cluster 1, again raised the possibility of cross contamination of the Drop-seq data by >1 retinal cell type (Fig. 6). Interestingly, there was no overlap in the expression of RBC genes in rod photoreceptors, the main pre-synaptic partner for RBCs in the scotopic visual pathway, except for a very low level of expression of *Prkca*. Analogous comparisons with the Drop-seq profiling data of different bipolar subtypes published by Shekhar *et al*.^18^ yielded similar results (data not shown)^12^.

### *Car8* is a strong candidate gene for direct regulation by BHLHE23

**Figure 7:**
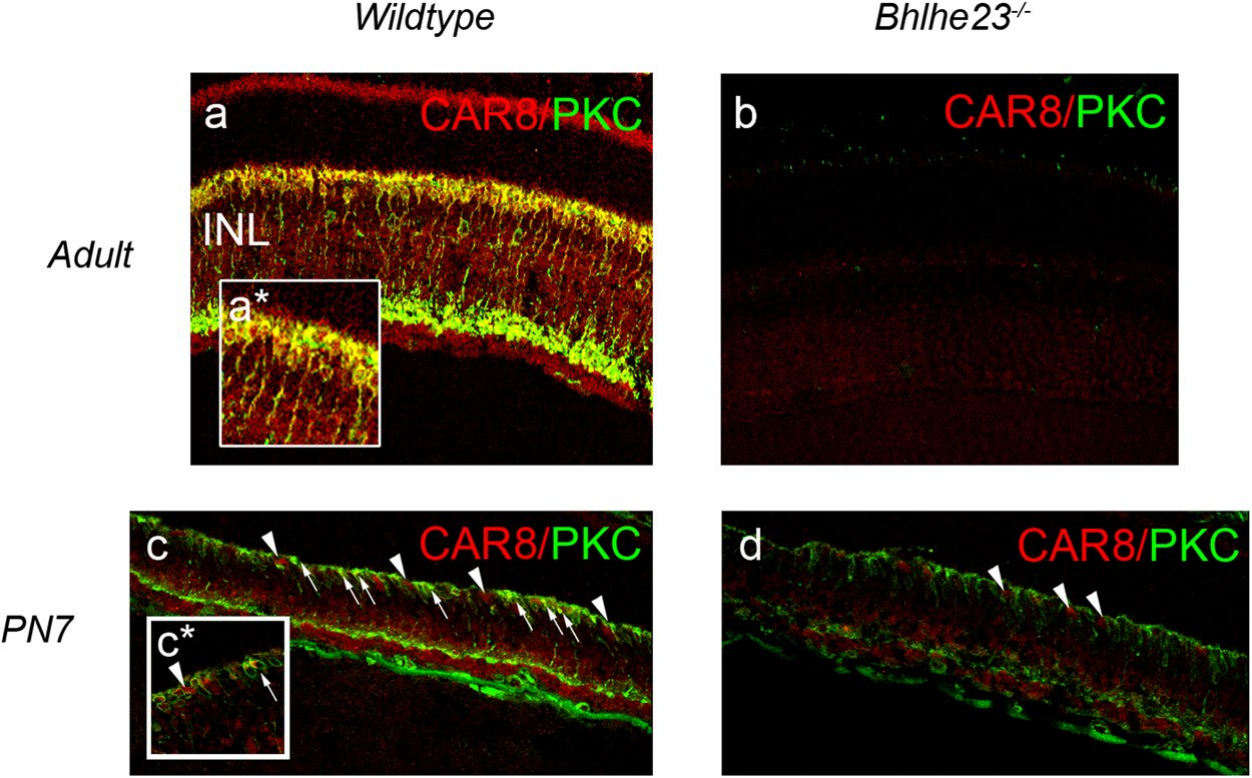
*Car8* is a strong candidate gene for direct regulation by BHLHE23. Representative confocal micrographs of retinal sections stained with antibodies to CAR8 and the RBC marker, PKC. **(a)** As in previous reports, CAR8 was expressed in PKC^+^ RBC bodies in adult wild type (WT) retina. **(b)** CAR8 was absent from adult *Bhlhe23*^−/−^ retina. **(c)** At PN7, the majority of PKC^+^ RBCs expressed CAR8 (arrows) in WT retina and we also observed a small number of CAR8^+^ cells that did not co-express PKC (arrowheads). Their position, in the outermost inner nuclear layer, suggested they were CBCs. **(d)** In the *Bhlhe23*^−/−^ retina at PN7, there appeared to be fewer PKC^+^ RBCs present. In those PKC^+^ RBCs that were still present, the level of CAR8 expression was markedly reduced/absent. Moreover, single positive CAR8^+^ cells persisted in the *Bhlhe23*^−/−^ retina which appeared to be CBCs from their position and morphology (arrowheads). These data strongly suggest that *Car8* is directly regulated by BHLHE23 in RBCs.

There was very little overlap in the transcriptome of the developing *Bhlhe23*^−/−^ retina at PN7 compared with the 3 published adult RBC transcriptomes we compared in this study^16–18^ and our own adult RBC transcriptome derived from the adult *Bhlhe23*^−/−^ retina (Supplementary Table S1). Indeed, the expression of only two genes were significantly different to WT in the PN7 and adult *Bhlhe23*^−/−^ retina with p_adj_ value < 0.05: *Car8* (down 1.93 fold, p_adj_ = 1.25 × 10^−5^ [PN7]; down 7.06 fold, p_adj_ = 2.21 × 10^−8^ [adult]) and *Il1rap* (down 1.39 fold, p_adj_ = 0.031 [PN7]; down 4.72 fold, p_adj_ = 3.90 × 10^−8^ [adult]). The expression of *Car8* in adult mouse retina had been described previously^24^, but we were interested in this gene as a target for BHLHE23 regulation because *Car8* is expressed in RBCs^15^, and we had found that the expression of *Car8* transcript was significantly different in the adult and developing *Bhlhe23*^−/−^ retina with large fold changes. We confirmed that CAR8 was expressed in the nuclei of RBCs and that all PKC^+^ cells were also CAR8^+^ in adult WT retina (Fig. 7a). At PN7 in WT retina, we observed that all developing PKC^+^ RBCs expressed CAR8, but that there were also a small number of CAR8^+^ cells that did not co-express PKC (Fig. 7c). The position of these cells in the INL suggested they were CBCs. We found that the number of PKC^+^ RBCs was reduced in the *Bhlhe23*^−/−^ retina at PN7 although the level of PKC expression in those remaining RBCs was unchanged (Fig. 7d). In contrast, the level of CAR8 expression in those remaining PKC^+^ RBCs was much reduced/absent while single positive CAR8^+^ cells (presumed CBCs) persisted in the *Bhlhe23*^−/−^ retina. These data strongly suggest that *Car8* is directly regulated by BHLHE23 in RBCs.

## Discussion

Classic linkage studies in families affected by night blindness as well as recent advances in whole exome sequencing have furthered the discovery of >60 genes associated with progressive night blindness or RP^25^. In comparison, seventeen genes have been linked to CSNB, a group of retinal disorders that usually cause non-progressive night blindness^3^. Indeed, for a small minority of genes, e.g. *RHO*, *PDE6B* and *GNAT1*, certain mutations result in RP, and others result in CSNB^4–8^. Yet, despite these advances in gene discovery, there remains a significant proportion of patients in whom a causal mutation has yet to be identified. In these patients, the identification of a genetic variant as causal for their disease depends on the filtering of their DNA sequencing results through a pipeline that checks any identified variants which co-segregate with the disease with: (i) databases of normal variants in the population; (ii) programs that predict the likely pathogenicity of novel variants, and (iii) retinal expression data from available transcriptomic databases^3^. It would also depend on accurate phenotyping, e.g. by non-invasively measuring the thickness of individual retinal layers, including the INL, by optical coherence tomography (OCT). We would predict that patients with cellular defects of the RBC population would have an electronegative scotopic ERG and a thinner INL on OCT; while other patients, in whom RBCs are dysfunctional but still present, might only have an electronegative scotopic ERG phenotype. Since relatively few of the genes we identified in the RBC transcriptome were exclusively expressed by RBCs, it is likely that mutations of these genes will affect other retinal cell types as well. Consequently, the robust identification of the RBC transcriptome will facilitate genetic screening programs of patients with CSNB with their characteristic electronegative ERG phenotype, as well as proposing candidate genes for further research into mechanisms underlying mammalian night vision.

Other groups have similarly sought to identify genes implicated in inherited causes of night blindness by profiling the transcriptomes of FACS sorted *Nrl*p-GFP labelled rods^26^ or the retinal transcriptomes of mouse models that lack rod photoreceptors^27,28^. Since rod photoreceptors are the most prevalent cell type in the retina, genes expressed in rods that are implicated in night blindness, e.g. *Rho* and *Gnat1*, have been identified as the most abundantly expressed genes in whole retinal samples from wild type mice^29^. Hence, there has been a drive to identify retinal transcripts from whole retinal samples and across different developmental timepoints, but without necessarily determining the cell-type specific expression of these transcripts^30^. Exciting technological advances in profiling the genetic signatures of individual cell types have provided invaluable insights into the neurogenetic architecture of the mammalian retina^17^ and brought the prospect of determining the RBC transcriptome within reach. To this end, we investigated the overlap between RBC transcriptomes derived from published retinal expression data with our own^16–18^. Only 22 markers (Fig. 4b, Supplementary Table S1) were common to all 4 adult RBC transcriptomes (including our own). The variation between studies is not surprising considering the varied methods used by each. For example, the strength of the Drop-seq method^17,18^ is the ability to profile gene expression in tens of thousands of cells in parallel, capturing the diversity of cells in the retina, and the two studies which used Drop-seq showed the greatest overlap in their RBC transcriptomes (63/64 genes identified by Macosko *et al*.^17^) (Fig. 4a). However, this comes at the cost of low sequencing coverage per cell (median of 744 reads mapped per cell) meaning that some genes with low expression, e.g. *Iltifb*, may not be detected. Moreover, it is possible that some clusters were contaminated by >1 retinal cell type, given the widespread presence of *Prkca*, *Car8* and *Bhlhe23* transcripts in multiple retinal cell clusters (Fig. 6)^18^. This is important, since one of these studies proposed the existence of two additional putative CBC subtypes based on their gene profiling results^18^.

Our approach was to take advantage of the *Bhlhe23*^−/−^ mouse retina to identify the RBC transcriptome, since the *Bhlhe23*^−/−^ mouse retina has a specific cellular defect in RBCs^11^. As the expression of *Bhlhe23* is almost entirely specific to RBCs in adult retina^11^, the majority of genes we have identified as significantly down-regulated in the adult *Bhlhe23*^−/−^ retina will be due to the absence of RBCs. Indeed, 74/84 genes we identified overlapped with at least one other RBC transcriptome and showed the greatest similarity with the data from the two studies which sorted bipolar cells from other retinal cell types using *Vsx2*-GFP and *Pcp2* reporter mouselines^16,18^.

Consistent with the importance of RBCs in rod signalling pathways, the largest clusters of differentially expressed genes in the PN7 and adult *Bhlhe23*^−/−^ retina were involved in synaptic signalling, structure or function (Figs 1 and 3). Yet it was striking that there was little overlap between the identities of these genes in functional enrichment analyses of the developing and adult *Bhlhe23*^−/−^ retinal transcriptomes, except for *Prkca*. Predictably, there were more differentially expressed genes involved in regulating gene transcription/translation and the cell cycle at the earlier time point. At PN7, mouse eyes have yet to open and rod-RBC synapses are still forming and maturing. It is likely that rod-RBC signalling has a different role during retinal development; perhaps a neurotrophic function to preserve the RBCs that successfully connect with rod photoreceptor terminals from developmental pruning. PKC is known to accelerate glutamate-driven signal transduction and termination in RBCs of the adult retina^31^, but its role during development is not known. Elsewhere in the CNS, PKC isoforms are expressed in developing synapses and their expression patterns change as these synapses mature, suggesting that PKC influences synaptogenesis^32^. It is possible that PKC has a similar role in the synaptogenesis of developing RBCs in the retina.

We have confirmed that a number of proteins encoded by genes in the RBC transcriptome localise to bipolar cell dendrites and/or axon terminals, e.g. LRP2BP, SLCO5A1, LRRTM4, and MYPN, consistent with a putative role in synaptic signalling or structure. Although the expression patterns of these proteins are not all specific to RBCs, they are likely to have a greater impact on night vision than photopic vision, purely because the majority of bipolar cells in the retina are RBCs. This is the most likely explanation for gene defects in *GRM6*, *TRPM1* and *LRIT3* causing more problems with night vision than photopic vision, even though they are expressed by all types of ON-bipolar cells in the retina^3^, and it would be interesting to investigate whether LRP2BP, SLCO5A1, LRRTM4, or MYPN co-localise with GRM6, TRPM1 or LRIT3 in rod bipolar cell dendrites in future studies.

LRP2BP is a scaffold protein known to bind and recruit proteins to the megalin receptor that participates in endocytosis and signal transduction^33^. Intriguingly, one of the binding partners of LRP2BP is the PKCα-binding protein (PICK1)^34^, and so it is possible that LRP2BP may be involved in rod-RBC signal transduction via its interactions with megalin and PICK1 in the retina. LRRTM4 is a member of the LRR family of proteins that are known to be involved in synaptic development and function. LRRTM4 has been shown to regulate excitatory synapse development in cultured neurons by binding the receptor, Glypican^35^, a protein that is also expressed by ON-bipolar cells^20^, suggesting that LRRTM4 might interact with Glypican in the retina to regulate signal transduction in RBCs. There is little relevant knowledge for the function of SLCO5A1 or MYPN in the retina, although SLCO5A1 is a membrane protein that is expressed in foetal and adult brain^36^. Over-expression of SLCO5A1 in HeLa cells leads to the differential expression of genes involved in synapse assembly and organisation^37^. MYPN, on the other hand, is known only to be expressed in heart and skeletal muscle where it interacts with α-actinin to form an anchor for actin filaments^38^. It is possible that SLCO5A1 and MYPN have similar structural roles in synaptic assembly in the retina.

In contrast to the expression of LRP2BP, SLCO5A1, LRRTM4, and MYPN in bipolar cell dendrites and/or axon terminals, we found that ILTIF was expressed in the cell bodies of RBCs, CBCs and ACs. ILTIFB (also known as IL22B) shares 98% identity with ILTIFA (IL22) and 22% identity with IL10^39^. It is a cytokine that is expressed constitutively in brain and can induce STAT activation in neuronal cell lines^39^. This may be relevant since STAT3-mediated signalling regulates rod photoreceptor development and STAT3 activation is, in turn, regulated by PKC^40^.

In contrast to one previous study^18^, we found that IL1RAP did not co-localise with PKC^+^ RBCs. Instead, IL1RAP appeared to be more highly expressed in ACs. IL1RAP functions as a trans-synaptic adhesion molecule in neurons that can induce post-synaptic neuronal differentiation through its interaction with receptor Protein Tyrosine Phosphatase δ (PTPδ)^41^. *Il1rap* was one of the most significantly differentially expressed genes at PN7 and in adult *Bhlhe23*^−/−^ retina, and though IL1RAP was not expressed at detectable levels in RBCs, it is possible that dysregulation of this trans-synaptic regulator of neuronal differentiation occurred indirectly in ACs due to the loss of RBCs. Less is known about WSCD2 (also expressed by ACs), though in yeast, the WSC domain appears to function in the PKC1-MPK1 signalling response to environmental stress^42^. It is conceivable that the function of WSCD2 is conserved in mammalian cells to act in homologous PKC-MAPK signalling pathways.

*Car8* is a gene that was already known to be expressed in developing and mature RBCs^15,24^. CAR8 encodes a catalytically silent carbonic anhydrase^43^. Though it is known to be expressed in cerebellar Purkinje cells, hippocampus, cerebral cortex and thalamus, and may be involved in vesicular transport, synaptic transmission and synaptogenesis, its function remains largely unknown^43–45^. In the mouse retina, CAR8 is expressed in RBCs and a small subset of CBCs and ACs^24^. Null mutations of the *Car8* gene do not appear to affect RBC density, synaptic organisation or rod-RBC signal transduction, but do affect light-evoked AII amacrine responses^24^. Hence, CAR8 appears to affect RBC-AII AC signal transduction in the scotopic signalling pathway. We were particularly interested in this gene because *Car8* was one of the most significantly down-regulated genes in both the PN7 and adult *Bhlhe23*^−/−^ retina. We found that CAR8 expression was markedly reduced/absent in PKC^+^ RBCs in the developing *Bhlhe23*^−/−^ retina at PN7, strongly suggesting that *Car8* is directly regulated by BHLHE23 at a time when RBC signal transmission via ACs to ganglion cells is first observed^46^. Though BHLHE23 is known to be one of a very large family of basic helix-loop-helix transcription factors^47^, little is known about the genes that BHLHE23 regulates. Further investigation of genes directly targeted by BHLHE23 is limited by the lack of a suitable antibody for chromatin precipitation studies and would be beyond the scope of this paper. Yet, further studies of the direct targets of BHLHE23 regulation would prove very valuable in future, once a suitable antibody becomes available.

In summary, we have derived a consensus for the RBC transcriptome in the adult mouse retina. By combining data from 4 independent gene expression profiling experiments, our approach is less vulnerable to the weaknesses of each individual study. We have found that the RBC transcriptome is comprised mainly of genes that are involved in synaptic signalling, assembly and structure and that the expression pattern of several novel RBC gene products is consistent with their putative roles in RBC signalling. These findings ought to facilitate genetic screening programs of patients with electronegative ERGs from inherited causes of night blindness, as well as proposing candidate genes for further research into mechanisms underlying mammalian night vision.

## Methods

### Husbandry

Targeting of the *Bhlhe23* gene and genotyping of the *Bhlhe23* knock out allele was previously described by Kim *et al*.^15^. The *Bhlhe23* knock out colony was established in the Animal Service Unit at the University of Bristol and housed under specific pathogen-free conditions with food and water provided *ad libitum*. The use of animals and experimental protocols were approved by the University of Bristol’s Animal Welfare and Ethical Review Body (AWERB), met with University of Bristol institutional guidelines and adhered to the Animals (Scientific Procedures) Act 1986 issued by the Home Office and the ARVO Statement for the Use of Animals in Ophthalmic and Vision Research.

### RNA preparation

Retinas were dissected in ice-cold PBS and stored in TRIzol® (Thermo Fisher Scientific, Waltham, MA, USA) or RNAlater® (Qiagen) at -20 °C. RNA extraction and purification was performed using the RNeasy^®^ Mini kit from Qiagen (Hildern, Germany) before quantification on a Nanodrop spectrophotometer (ND-1000, Thermo Fisher Scientific).

### Gene expression microarray analyses

RNA (250 ng) from 4 sex and litter-matched adult pairs of *Bhlhe23*^−/−^ and WT mice was prepared for hybridisation onto Affymetrix GeneChip® Mouse Genome 430 2.0 Arrays using GeneChip® 3′ IVT Express kits following manufacturer’s guidelines (Affymetrix, Santa Clara, CA, USA). Hybridised arrays were washed, stained and scanned using the Affymetrix Fluidics Station 450 and GeneChip® Scanner 3000 using Affymetrix GeneChip® Command Console® instrument control software. Probe set signal intensities were combined to give probe expression levels using the GCRMA (Guanine-cytosine content adjusted robust multi-array average) method. The bottom 50% of probes with the lowest variance across samples was removed from further analyses. Differential gene expression between conditions was examined by linear regression using limma^48^. A multiple testing correction was performed using the Benjamini and Hochberg (BH) procedure to adjust p-values^49^.

### RNA-Seq analyses

RNA (1 ug) from 6 pairs of litter-matched *Bhlhe23*^−/−^ and WT mice at PN7 was quantified and checked for purity with an Agilent Bioanalyser 2100 using the Agilent DNA 1000 chip (Agilent, Santa Clara, CA, USA) prior to the preparation of barcoded sequencing libraries using the Illumina® TruSeq Stranded Total RNA Low-Throughput sample RiboZero Human/Rat/Mouse prep kit following manufacturer’s guidelines (Illumina, San Diego, CA, USA). Libraries were quantified with a Qubit® fluorometer (Thermo Fisher Scientific) using the double stranded DNA High sensitivity assay. 10 nM of each library was pooled, and a final dilution of 9pM was sequenced (100 bp paired end run) on an Illumina HiSeq. 2500 (Illumina). Low quality bases and adapter sequences were trimmed from reads using Trimmomatic^50^. Reads were mapped to the GRCm38.p3 mouse reference genome^51^ with TopHat2^52^. HTSeq-count^53^ was used to count the number of reads aligned to each feature specified in Ensembl gene set annotation files. Differential expression between conditions was analysed by negative-binomial regression using DESeq2^54^. Samples were not sexed prior to experimentation. Post-hoc inference of sex, by comparison of *Inactive X specific transcript* (*Xist*) expression across samples, revealed that four of six mutant and three of six WT samples were female (Supplementary Table 1, Sheet 2, row 3528). Consequently, sex was included as a fixed-effect covariate in the differential expression analysis and the segregation of sex in the samples is illustrated in Supplementary Figure S2b. Since *Xist* was not differentially expressed between conditions (up 1.13 fold, nominal p-value = 0.20), this adjustment for imputed sex did not appear to bias the results. P-values were adjusted by the Benjamini an Hochberg procedure.

### Derivation of a consensus adult rod bipolar transcriptome

The rod bipolar transcriptome had previously been investigated in three different studies, each using different experimental protocols:

#### Drop-seq gene profiling of retinal cell types

Macosko *et al*.^17^ used Drop-seq to profile the transcriptomes of 44,808 mouse retinal cells at PN14^17^. The cells were clustered into 39 distinct populations, including eight bipolar cell sub-types. Gene expression counts for each^17^ cell were obtained from Gene Expression Omnibus (GSE63472). Cells were grouped by assigned cluster (available: http://mccarrolllab.com/wp-content/uploads/2015/05/retina_clusteridentities.txt [accessed 21/9/16]) and the proportion of cells expressing each gene was calculated. The cluster showing high expression of *Bhlhe23* and *Prkca* (‘Bipolar: 1′/cluster 26) was assumed to represent RBCs (Fig. 7). Cluster 26 contained 2,217 cells and the remaining clusters contained 42,591 cells. Using the methods described in this study for differential gene expression analysis, 87 genes were found to be differentially expressed in RBCs (cluster 26) compared to other cell types in the experiment (Supplementary Table S1)^17^. Of these differentially expressed RBC genes, 64 that were overly-represented were taken to be RBC specific. A heatmap showing the portion of cells within each cluster that expressed the genes of interest was produced using ggplot2. http://mccarrolllab.com/wp-content/uploads/2015/05/retina_clusteridentities.txt [accessed 21/9/16]) and the proportion of cells expressing each gene was calculated. The cluster showing high expression of *Bhlhe23* and *Prkca* (‘Bipolar: 1′/cluster 26) was assumed to represent RBCs (Fig. 7). Cluster 26 contained 2,217 cells and the remaining clusters contained 42,591 cells. Using the methods described in this study for differential gene expression analysis, 87 genes were found to be differentially expressed in RBCs (cluster 26) compared to other cell types in the experiment (Supplementary Table S1). Of these differentially expressed RBC genes, 64 that were overly-represented were taken to be RBC specific. A heatmap showing the portion of cells within each cluster that expressed the genes of interest was produced using ggplot2.

#### Drop-seq gene profiling of bipolar cell types

Shekhar *et al*.^18^ used a transgenic line^18^ of mice expressing *Vsx2*-GFP, a reporter line which labels bipolar cells and Müller glia, and used FACS to sort ~25,000 cells from a suspension of retinal cells at PN17^18^. The transcriptomes of these cells were then profiled using Drop-seq and clustered into 26 cell types. Processed data files were accessed from the Gene Expression Omnibus (GSE81905). High expression of *Prkca* and the presence of *Bhlhe23* in cluster 1 identified it as representing RBCs. Code provided by the authors (available: https://github.com/broadinstitute/BipolarCell2016 [accessed: 21/9/16]) was adapted to extract genes differentially expressed in RBCs (cluster 1) compared to other cell types^18^.

#### Microarray gene profiling of retinal cells using reporter mouselines

Siegert *et al*.^16^ used 22 transgenic mouse^16^ lines, each with different fluorescent reporters to label retinal cell types. These fluorescent proteins were used to sort the cells with FACS, and mRNA expression was quantified using Affymetrix GeneChip^TM^ Mouse Exon 1.0 ST arrays. The transgenic line expressing *Purkinje cell protein 2-eGFP* (PCP2-eGFP) was used to identify FACS sorted RBCs. The results of these gene expression microarray experiments (performed in triplicate) are available from the Gene Expression Omnibus (GSE33085). Expression intensities were log transformed and the differential expression between PCP2-tagged cells (N = 3) compared to the average of all other FACS sorted cell types was calculated by linear regression using the Bioconductor package *limma*.

To generate a consensus adult RBC transcriptome, we combined four transcriptomes that included the following datasets:

i. Woods *et al*.: down-regulated genes in the adult *Bhlhe23*^−/−^ retina compared to WT, adjusted p-value (p_adj_) <0.05.
ii. Macosko *et al*.^17^: up-regulated genes in cluster 26 compared to other clusters, as defined by the authors^17^.
iii. Shekhar *et al*.^18^: up-regulated genes in cluster 1 compared to other clusters, p_adj_-values < 0.05^18^.
iv. Siegert *et al*.^16^: up-regulated genes in PCP2-tagged cells after FACS compared with other cells, p_adj_-values < 0.05^16^.

Only genes present in ≥2 transcriptomes were included in subsequent analyses.

### RT-PCR

RNA (1ug) from six pairs of *Bhlhe23*^−/−^ and litter-matched wild type mice at PN7 was reverse transcribed using the Qiagen Omniscript® RT kit. RT-PCR was performed using transcript specific primers (Supplementary Table S2) and the Qiagen QuantiFast SYBR Green kit following manufacturer’s guidelines. PCR products were quantified using the standard curve method. Matched pairs with expression values greater than three median absolute deviations from the mean were removed as outliers. Log-transformed values were compared using paired student *t*-tests.

### Functional enrichment analyses of differentially expressed genes

To increase our power to detect enriched ontologies and pathways, we accepted a false discovery rate adjusted p-value (p_adj_) of 0.25, since enrichment tests are still valid if the gene list contains false positives and because the inclusion of more (random) false positive terms would attenuate the enrichment test statistics in an unbiased way (bias towards the null). Differentially expressed genes with p_adj_ value < 0.25 were analysed for enrichment of gene ontologies using ClueGO (v2.1.7)^55^ for Cytoscape (v3)^56^, GO_Biological Processes (11.09.2015)^57^, GO_Cellular Components (11.09.2015)^57^, KEGG (14.09.2015)^58,59^ and REACTOME pathways (14.09.2015)^60^. Only classes containing 3 or more genes were tested. Enrichment statistics were calculated using a right-sided hypergeometric test with Bonferroni step down correction^55^.

### Immunolabelling

Eye-cups were dissected in ice-cold PBS, fixed in 4% paraformaldehyde in PBS for 1-2 hours at 4 °C, then cryoprotected in 30% sucrose in PBS before storage in OCT (Thermo Fisher Scientific) at −80 °C. Frozen tissue was cut into 12 µm sections prior to washes in PBS, permeabilization with 0.1% Triton-X in PBS (PBST), and blocking in 10% normal serum in PBS for one hour in a humid chamber. Primary antibodies were diluted in blocking buffer (Supplementary Table S3) and incubated with sections overnight at 4 °C. The following day, tissues were washed in PBS before secondary antibodies (Invitrogen™|Thermo Fisher Scientific) diluted 1:500 in blocking buffer were added and incubated at room temperature for one hour, followed by further washes in PBS. Coverslips were mounted with hard set Vectashield® containing DAPI (Vector Laboratories Inc., Burlingame, CA, USA). For double-labelling, antibodies were applied concurrently during both primary and secondary labelling steps. Images were obtained using a Leica Laser Scanning Confocal microscope and processed using the Leica LAS AF software (Leica, Wetzlar, Germany).

### Data availability statement

The data generated and analysed in this study are available in ArrayExpress (https://www.ebi.ac.uk/arrayexpress/) under accession number E-MTAB-6069 (microarray) and E-MTAB-6071 (RNAseq), and in this published article and its Supplementary Information files. Publically available datasets were accessed from the Gene Expression Omnibus as indicated in the Methods above.

## Electronic supplementary material

Supplementary Information

Supplementary Table S1
