# Supplementary material for: A comparative analysis of rod bipolar cell transcriptomes identifies novel genes implicated in night vision
Source: Sci Rep. 2018 Apr 3;8:5506. doi: 10.1038/s41598-018-23901-6 (PMC5883057; doi:10.1038/s41598-018-23901-6)
Supplement: Supplementary file 1 — Supplementary Information [file 41598_2018_23901_MOESM1_ESM.docx]

**A comparative analysis of rod bipolar cell transcriptomes identifies novel genes implicated in night vision**

**Sasha M. Woods, Edward Mountjoy, Duncan Muir, Sarah E. Ross, Denize Atan**

**Supplementary Information**

**Supplementary Tables**

**Table S1.** RBC gene sets derived from: 1. A comparison of the transcriptomes of adult *Bhlhe23^-/-^* retina vs wild type (WT) (n=4) using microarrays. Genes known to be expressed in RBCs are highlighted in green. Genes expressed in, but not specific to, RBCs are highlighted in orange (Sheet 1); 2. A comparison of differentially expressed genes in *Bhlhe23^-/-^* retina vs WT at postnatal day 7 (PN7) (n=6) using RNA-seq. Genes with adjusted p-value <0.05 are highlighted in yellow (Sheet 2); 3. Genes identified from Drop-seq data of different cell types in whole retina^11^ using *Prkca* transcript as an identifier of RBCs (Sheet 3); 4. Genes identified from Drop-seq data of *Vsx2-*GFP*^+^* cells in whole retina^12^ using *Prkca* transcript as an identifier of RBCs (Sheet 4); 5. Genes identified from microarray data used to profile *Pcp2^+^* cells^9^ (Sheet 5). The overlap between gene sets in Sheets 1, 3, 4, and 5 with P_adj_ <0.05 is shown in Sheet 6 and Figure 3.

| Name | Sequence (5’-3’) |
| --- | --- |
| *Gapdh* | Forward: TTCACCACCATGGAGAAGGC  Reverse: GGCATGGACTGTGGTCATGA |
| *A230077H06Rik* | Forward: CTGGGCATGGTGGTATAAATGTC  Reverse: GCTGTGTCCAAGCCAAACAA |
| *Car8* | Forward: GTGGGGTTACGAGGAAGGTG  Reverse: TCCAGCAGTGAGGGGTCATA |
| *Il1rap* | Forward: AATTTGTGCTGCTGACGCTG  Reverse: GTGACAATTCCCCCAGGCAG |
| *Gm11619* | Forward: CGGCTCTAGGAAGAGATGCAAG  Reverse: GCCGAGCAGAATGAAACGG |
| *Slco5a1* | Forward: GTCAGAGACCTACCAAGAGCAG  Reverse: TGTGACTCGATGAACTTGGGG |

**Table S2. Primer sequences for RT-PCR**

| Antibody | Dilution | Source | Catalogue number |
| --- | --- | --- | --- |
| Rabbit anti-CAR8 | 1:50 | Santa Cruz | SC-67330 |
| Rabbit anti-IL1RAP | 1:500 | Abcam | AB8110 |
| Rabbit anti-ILTIF | 1:100 | Abcam | AB18499 |
| Mouse anti-GAD65 | 1:500 | BD Biosciences | 559931 |
| Rabbit anti-GAD65 | 1:10,000 | T.M. Jessell |  |
| Goat anti-LRP2BP | 1:100 | Santa Cruz | SC-132741 |
| Sheep anti-LRRTM4 | 1:100 | R&D | AF5377 |
| Goat anti-MYPN | 1:500 | Santa Cruz | SC-79638 |
| Mouse anti-PKCα | 1:100 | Sigma-Aldrich | P5704 |
| Rabbit anti-PKCα | 1:10,000 | Sigma-Aldrich | P4334 |
| Mouse anti-RIBEYE | 1:10,000 | BD Biosciences | 612044 |
| Rabbit anti-SLCO5A1 | 1:100 | Abcam | AB1911412 |
| Goat anti-WSCD2 | 1:500 | Santa Cruz | SC-132741 |

**Table S3. Primary antibodies and their dilutions**

**Supplementary Figures**

| **a**  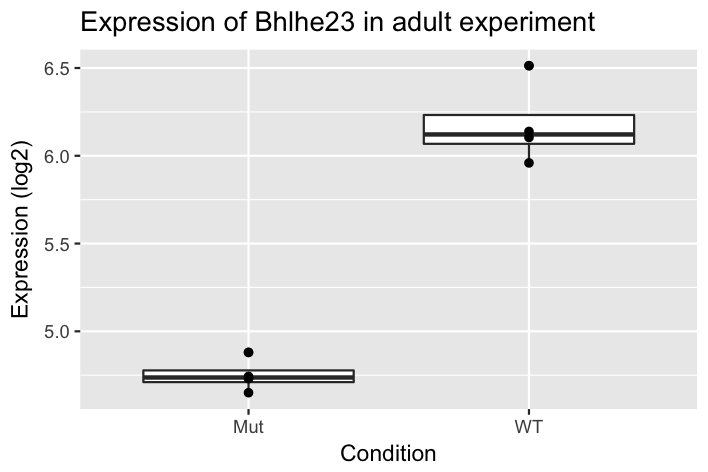 | **b**  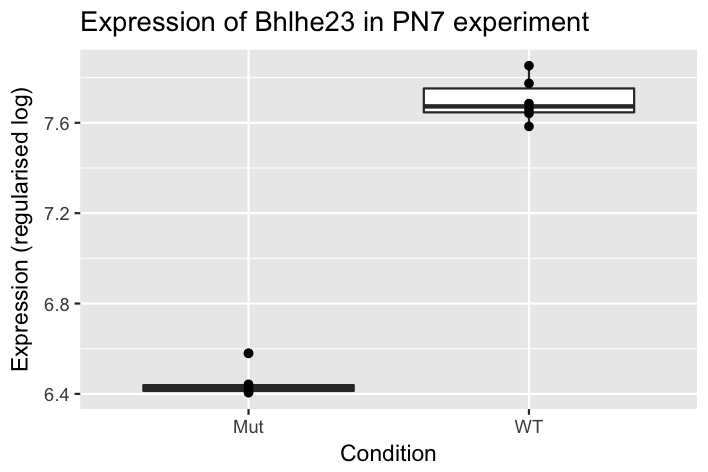 |
| --- | --- |

**Figure S1.** **Box plots of *Bhlhe23* expression in wild type (WT) and *Bhlhe23^-/-^* samples**. **(a)** *Bhlhe23* expression in adult *Bhlhe23^-/-^* retina vs WT profiled with gene expression microarrays. **(b)** *Bhlhe23* expression in *Bhlhe23^-/-^* retina vs WT at post-natal day 7 (PN7), profiled using RNA-seq. Regularised transformations for the RNA-seq data were blinded to condition. Both plots show that *Bhlhe23* expression was reduced in *Bhlhe23^-/-^* retinal samples compared to WT retinal samples.

| **a**  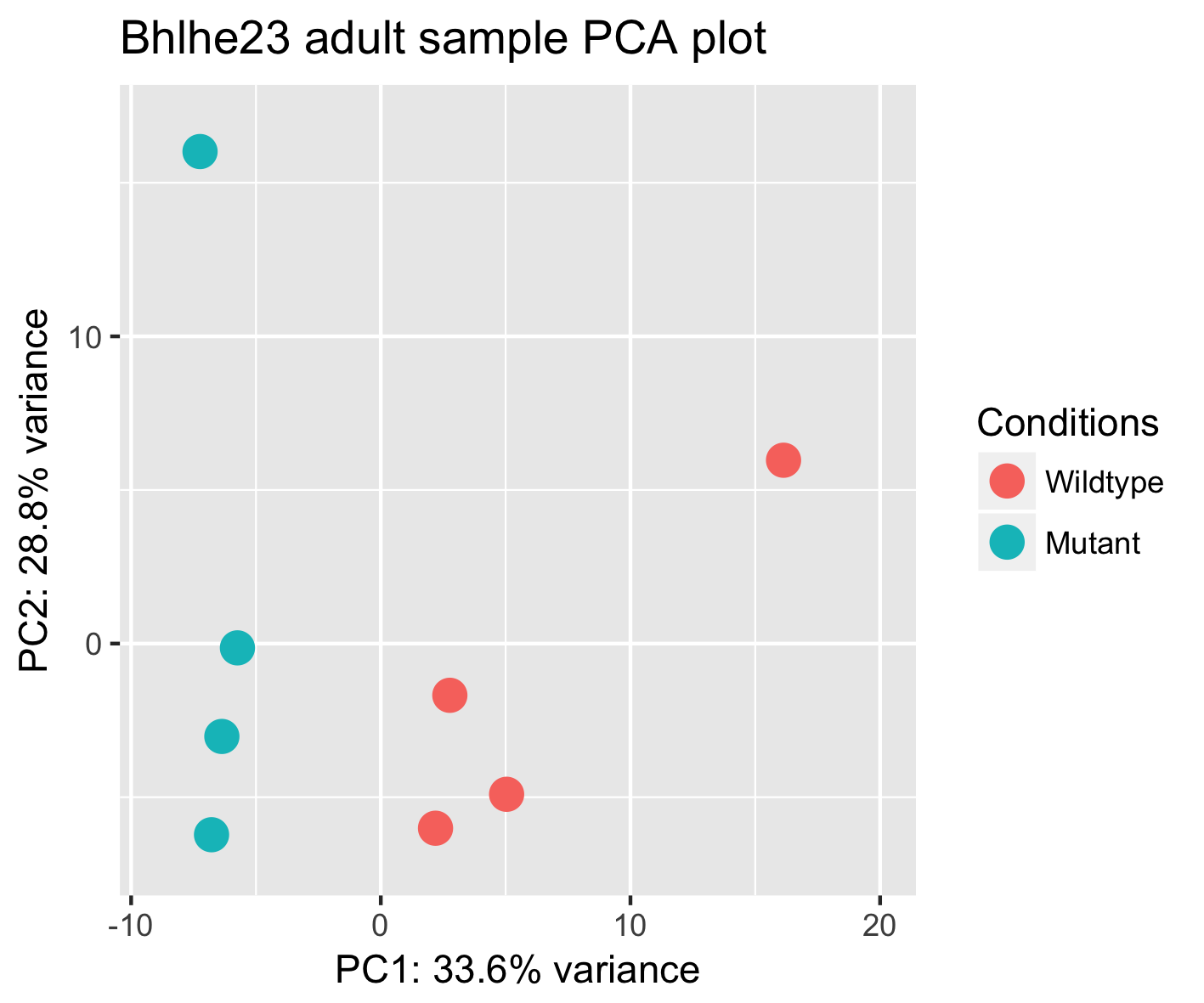 | **b**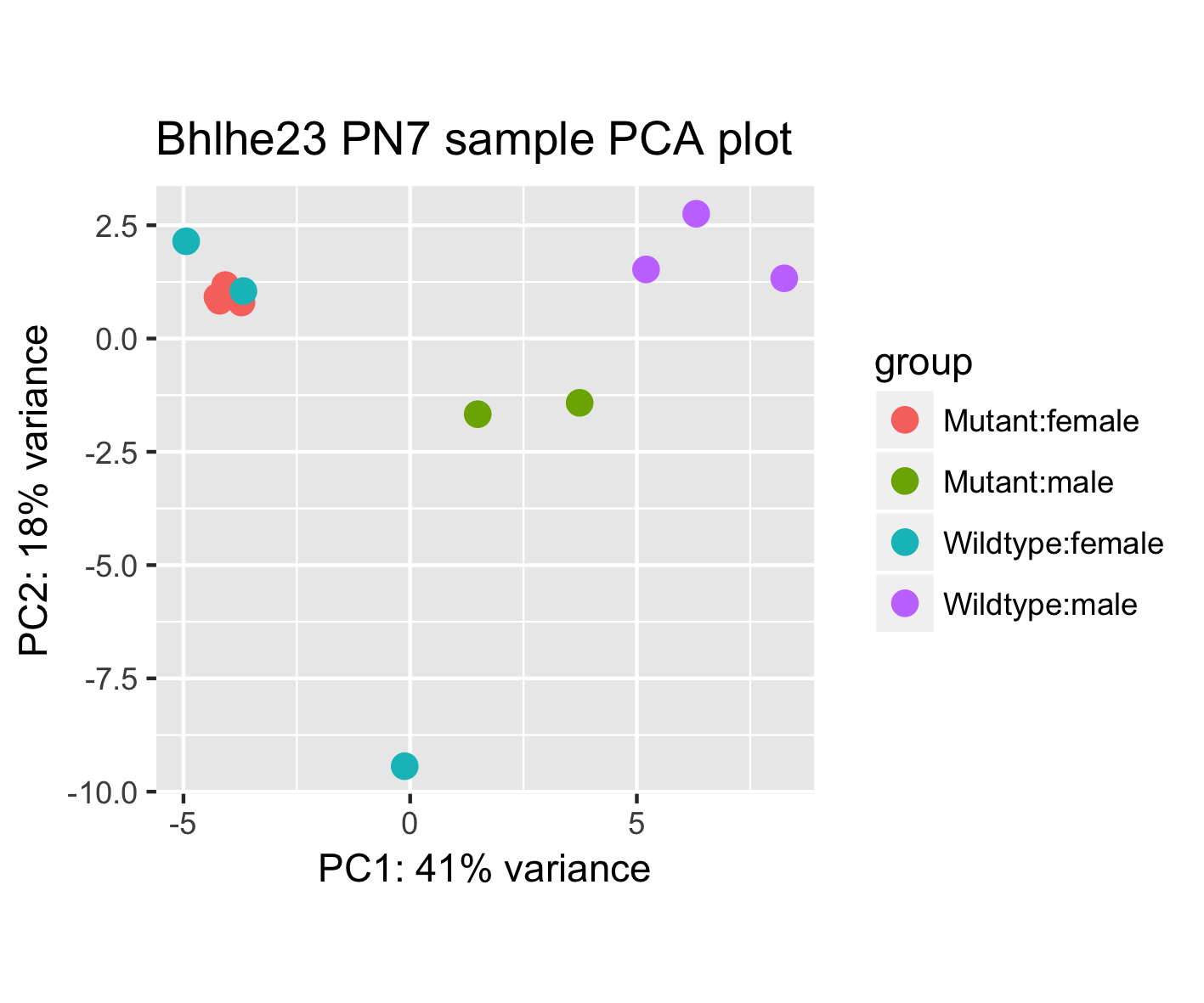 |
| --- | --- |

**Figure S2. Principal component analysis (PCA) plots showing the similarities between wild type (WT) and *Bhlhe23^-/-^* samples.** The first two principal components that explained the largest amount of variance are shown. Genes with the highest standard deviation across samples (n=500) were used as input. **(a)** In the adult microarray experiment, samples grouped by condition across PC1, showing that there were extensive differences in gene expression between the WT and *Bhlhe23^-/-^* samples. **(b)** In the postnatal day 7 (PN7) RNA-seq experiment, samples did not cluster distinctly based on condition. This likely represented the fact that there were few differences between WT and *Bhlhe23^-/-^* samples at PN7 since RBCs are lost from the *Bhlhe23^-/-^* retina from PN8 onwards (Bramblett *et al,* 2004). There was some grouping of samples by sex along PC1. Sex was therefore included as a fixed effects covariate in the differential expression analysis (see Methods).


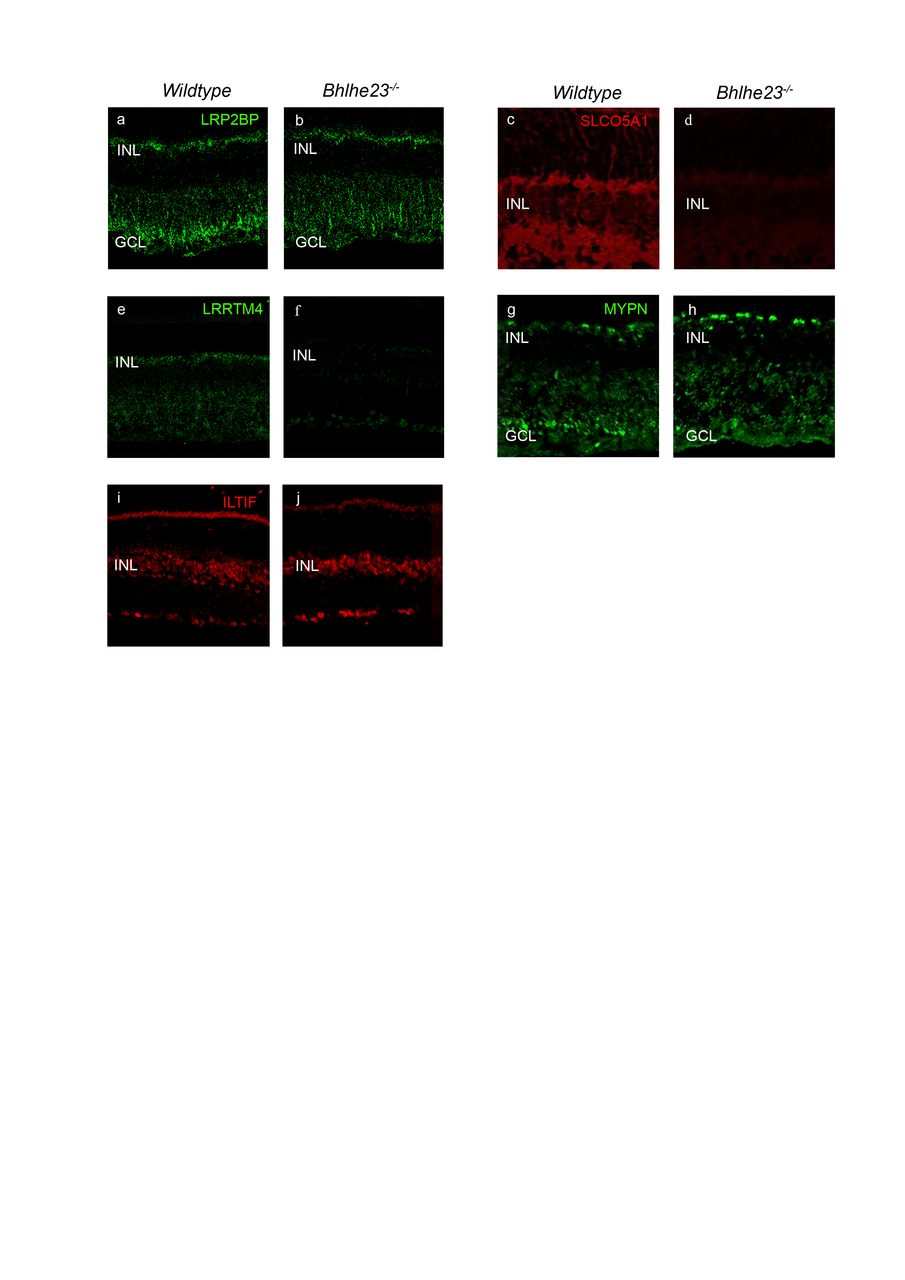


**Figure S3. Representative confocal micrographs of adult wild type (WT) and *Bhlhe23^-/-^* retinal sections.** **(a-b)** LRP2BP expression is reduced in *Bhlhe23^-/-^* retina compared to WT, particularly in axon terminals. **(c-d)** SLCO5A1 is dramatically reduced in *Bhlhe23^-/-^* retina compared to WT. **(e-f)** LRRTM4 is almost completely absent in *Bhlhe23^-/-^* retina compared to WT. **(g-h)** MYPN expression is absent from axon terminals in *Bhlhe23^-/-^* retina but persists in dendrites. **(i-j)** The number of ILTIF positive cells is reduced in *Bhlhe23^-/-^* retina.
